# Cheyne-Stokes Respiration in Patients with First-Ever Lacunar Stroke

**DOI:** 10.1155/2012/257890

**Published:** 2012-04-23

**Authors:** Marc Bonnin-Vilaplana, Adrià Arboix, Olga Parra, Luis García-Eroles, Josep M. Montserrat, Joan Massons

**Affiliations:** ^1^Service of Pneumology Division, Department of Neurology, Hospital Universitari del Sagrat Cor, Universitat de Barcelona, Viladomat 288, 08029 Barcelona, Spain; ^2^Cerebrovascular Division, Department of Neurology, Hospital Universitari del Sagrat Cor, Universitat de Barcelona, Viladomat 288, 08029 Barcelona, Spain; ^3^CIBER de Enfermedades Respiratorias (CibeRes-CB06/06), Instituto de Salud Carlos III, Sinesio Delgado 4, 28029 Madrid, Spain; ^4^Unidad de Organización, Planificación, y Sistemas de Información, Consorci Sanitari del Maresme, Carretera de Cirera s/n, 08034 Mataró, Spain; ^5^Sleep Unit, Service of Pneumology, Department of Medicine, Hospital Clínic, Universitat de Barcelona, Casanovas 143, 08036 Barcelona, Spain

## Abstract

The aim of this single-center prospective study was to assess the presence of Cheyne-Stokes respiration (CSR) and CSR-related variables in 68 consecutive patients with radiologically proven first-ever lacunar stroke undergoing a respiratory sleep study using a portable respiratory polygraph within the first 48 hours of stroke onset. CSR was diagnosed in 14 patients (20.6%). Patients with CSR as compared with those without CSR showed a significantly higher mean (standard deviation, SD) apnea-hypopnea index (AHI) (34.9 (21.7) versus 18.5 (14.4), *P* = 0.001) and central apnea index (13.1 (13.8) versus 1.8 (3.4), *P* = 0.0001) as well as higher scores of the Barthel index and the Canadian Neurological scale as a measure of stroke severity, and longer hospital stay. CSR was present in one of each five patients with lacunar stroke. The presence of CSR was associated with a trend towards a higher functional stroke severity and worse prognosis.

## 1. Introduction

Central sleep apnea and Cheyne-Stokes respiration are frequently observed during sleep in patients with stroke affecting large areas of the cerebral parenchyma [1–5] and in patients with congestive heart failure and low ventricular ejection fraction [6–10].

 In a previous study carried out in a nonselected sample of patients with cerebral infarction, Parra et al. [11] reported the presence of Cheyne-Stokes respiration in 26% of patients, a percentage higher than 6% of observed in the study of Bassetti and Aldrichet [12]. To date, Cheyne-Stokes respiration in stroke patients has been related to a worse prognosis probably because this abnormal breathing pattern is found in more extensive cerebral lesions and is also more common in hemorrhagic strokes than in ischemic infarctions [11, 13]. However, the traditional relationship between nocturnal Cheyne-Stokes respiration and large cerebral lesions is a matter of controversy given that Cheyne-Stokes respiration has been occasionally described in patients with transient ischemic attack (TIA) [11]. Lacunar infarctions are very homogeneous cerebral lesions regarding infarct size (maximal diameter of the lesion <20 mm), topography (affecting subcortical structures or the pons), and clinical features (limited neurological deficit and favorable neurological recovery on hospital discharge). In this respect, patients with lacunar stroke may constitute an intermediate group of stroke severity between TIA and extensive cardioembolic or atherothrombotic infarctions.

Given that the presence of Cheyne-Stokes respiration in patients with lacunar infarction has not been previously examined, a prospective study was designed. The objective of the study was to determine the frequency of Cheyne-Stokes respiration patients with first-ever lacunar infarction and to identify variables associated with this breathing pattern in this population.

## 2. Patients and Methods

The study population included 68 consecutive patients admitted to the Service of Neurology of Hospital Universitari Sagrat Cor in Barcelona (Spain) because of a first episode of a lacunar stroke. Lacunar infarcts were defined [14] as (a) sudden or gradual onset of a focal neurological deficit lasting >24 hours of the type described in the common lacunar syndromes (pure motor hemiparesis, pure sensory stroke, sensorimotor stroke, ataxic hemiparesis, dysarthria-clumsy hand, and atypical lacunar syndromes); (b) computed tomography (CT) scans or brain magnetic resonance imaging (MRI) was either normal or demonstrated only small, localized brain lesions with diameters <20 mm that seemed appropriate for the neurological deficits; (c) absence of cortical ischemia, cervical carotid, and/or vertebrobasilar stenosis (>50% diameter), or a major source for cardioembolic stroke. Patients with clinical symptoms of congestive heart failure or major cardiopathies were excluded from the study as were patients with a left ventricular ejection fraction ≤40% in the echocardiographic study. All eligible lacunar stroke patients underwent transthoracic echocardiography at the time of hospitalization, showing an ejection fraction >40%.

**Figure 1:**
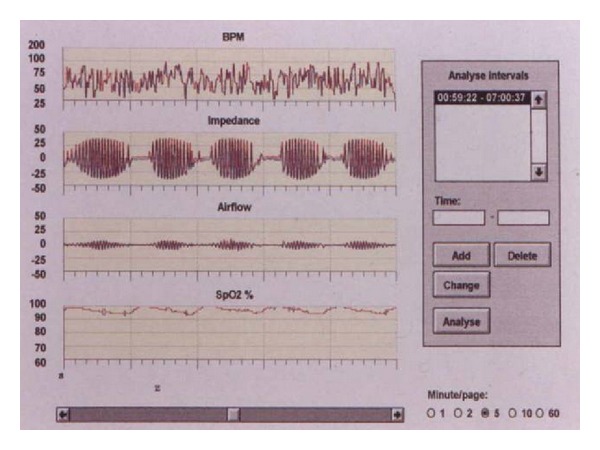
Cheyne-Stokes breathing in a patient with lacunar stroke. Central apnea with a crescendo/decrescendo pattern.

 In all cases, a respiratory sleep study was performed in the hospital ward during the first 48 hours after admission using a portable respiratory recording device (Hypno TT Digital Recorder) that has been previously validated using full polysomnography and used in stroke patients [11]. This portable device measures respiratory nasal flow (flow nasal sensory), chest wall movements (impedance), heart rate and thoracic impedance (ECG electrodes), arterial oxygen saturation (SaO_2_, finger pulse oximetry), and body position (position sensor). Sleep-related breathing disorders were classified as obstructive or central apnea. Central apneas were defined as a cessation of airflow for ≥10 s in the absence of any thoracic motion. A hypopnea was considered when a discernible reduction in airflow or thoracic motion that lasted >10 s and was associated with a cyclical dip in SaO_2_ of >3%. The Cheyne-Stokes respiration pattern was defined as a periodic breathing with central apnea in a crescendo/decrescendo pattern of >10% of the time spent in bed [2] (Figure 1). The apnea-hypopnea index (AHI) was calculated taking into account the time spent in bed with the respiratory recording device (lights out was considered the beginning of the recording and was usually initiated between 11:00 and 12:00 PM and terminated between 6:00 and 7:00 AM). Manual scoring of these variables was performed in all cases. An experienced scorer, who was blind to the clinical neurological data, performed the scoring. The percentage of nighttime with SaO_2_ of <90% (CT90) was obtained automatically.

 In all patients the following variables were recorded: age; sex; height; weight; body mass index (BMI); clinical features related to sleep-related breathing disorders, including snoring, observed apnea, and daytime sleepiness assessed by means of the Epworth sleepiness scale [15]; results of respiratory sleep studies; neurological and outcome data according to the standardized protocol of the Hospital of Sagrat Cor stroke registry. Definition of cardiovascular risk factors were those used by our group in previous studies [14, 16, 17]. All patients were admitted to the hospital within 48 hours of the onset of symptoms. Brain neuroimaging studies were performed within the first week of hospital admission. Patients with negative results in the first CT scan usually performed at the emergency department had a second CT examination during their stay in the hospital or were studied by MRI. Other investigations performed at the discretion of the attending physician included angio-MRI, echo Doppler of the supra-aortic trunks, arterial digital subtraction angiography, B mode echocardiography, and lumbar puncture.

 The Barthel index [18] was measured to assess performance in basic activities of daily living, total scores range from 0 (complete dependence) to 100 (complete independence). A Barthel index score of more than 75 indicated a good prognosis (absence of functional impairment or minimal functional disability at hospital discharge) [18]. The Canadian Neurological scale [19] was used to assess stroke severity; total score range from 0 (maximum impairment) to 10 (no impairment). The outcome was said to be good when the score was >7. Moreover, the modified Rankin scale [20] was used to assess clinical outcome at hospital discharge. We defined a good outcome as a modified Rankin scale ≥2.

 Prior to conducting the study, approval was obtained from the Ethical Committee on Clinical Research of the hospital. Written informed consent to undergo respiratory sleep studies was obtained from all patients.

### 2.1. Statistical Analysis

Univariate analysis for the different cardiovascular risk factors, clinical features, lacunar syndromes, respiratory data, topography of lacunar infarction, and scores of the Barthel index, Canadian Neurological scale, and modified Rankin scale in relation to the presence or absence of Cheyne-Stokes respiration was assessed with the analysis of variance (ANOVA) and the chi-square (*χ* ^2^) test with Yates or Bonferroni's correction when necessary. Statistical significance was set at *P* < 0.05.

## 3. Results

A total of 68 consecutive patients with lacunar infarction that was proven radiologically (CT and/or MRI) were included in the study. There were 31 men and 37 women, with a mean (standard deviation, SD) age of 73.2 (9.6) years and mean BMI of 26.3 (3.6) kg/m^2^.

Fourteen patients (20.6%) had Cheyne-Stokes respiration. There were 9 men and 5 women, with a mean age of 76.7 (6.8) years (range 39–89 years) and a mean BMI of 26.3 (4.3) kg/m^2^. The main cardiovascular risk factors were hypertension in 78.6% of the cases, diabetes mellitus in 28.6%, dyslipidemia in 28.6%, cigarette smoking in 28.6%, and peripheral artery disease in 7.1%. No case of decompensated congestive heart failure was recorded. Pure motor stroke was diagnosed in 6 patients, sensorimotor stroke in 3, dysarthria-clumsy hand plus ataxic hemiparesis in 3, and atypical lacunar syndrome in 2. Atypical lacunar syndromes included dysarthria associated with central facial palsy in 1 patient and isolated dysarthria in 1. The most frequent topographies of lacunes were the internal capsule and the pons in 35.7% of the patients each, the centrum semiovale in 21.4%, and the thalamus in 7.1%.

The mean (SD) score of the Epworth sleepiness scale was 3.3 (2.6) and the mean AHI 34.9 (21.7). The AHI was <10 in 1 patient, ≥10 in 13 patients, ≥20 in 12 patients, and ≥30 in 7 patients. A central apnea index >5 was observed in all 14 patients with Cheyne-Stokes respiration. The mean CT90 (percentage of time below 90% saturation) was 8.4 (11.3). The comparison between patients with (*n* = 14) and without (*n* = 54) Cheyne-Stokes respiration is shown in Table 1. Both groups were similar in relation to demographic data, clinical features, distribution of lacunar syndromes, and topography of lacunes. However, patients with Cheyne-Stokes respiration as compared with patients without sleep-disordered breathing showed significantly higher mean values of AHI (34.9 (21.7) versus 18.5 (14.4), *P* = 0.001) and central apnea index (13.1 (13.8) versus 1.8 (3.4), *P* = 0.0001). On the other hand, there was a trend towards higher functional impairment and worse prognosis in patients with Cheyne-Stokes respiration as noted by a lower percentage of patients with a score >75 of Barthel index (44.5% versus 68%, *P* = 0.397) and a score >7 of the Canadian Neurological scale (44.4% versus 75%, *P* = 0.21). Mean scores of the Barthel index, the Canadian Neurological scale, and the modified Rankin scale were similar in both groups (Table 2).

Mean scores of the Barthel index were 58.3 (32.5) for patients with Cheyne-Stokes respiration and 75.8 (27.9) for patients with normal breathing pattern (*P* = 0.130); the corresponding figures for the Canadian Neurological scale and the modified Rankin scale were 6.9 (1.9) versus 7.9 (2.1) (*P* = 0.131) and 2.4 (1.3) versus 2.2 (1.1), respectively.

None of the patients died within the first 30 days after stroke onset. The mean length of hospital stay was 14.9 (11.1) days in patients with Cheyne-Stokes respiration and 11.5 (5.6) in patients without Cheyne-Stokes respiration (*P* = 0.184).

## 4. Discussion

Cheyne-Stokes respiration has been usually described in patients with congestive heart failure [6–10] and in patients with extensive cerebral infarctions and poor outcome (3–5). Cheyne-Stokes respiration was also found to be associated with hypocapnia and poorer ejection fraction in a group of stroke patients (undifferentiated location) [7]. The present study also demonstrates that Cheyne-Stokes respiration is present in 20.6% of lacunar stroke patients with small size cerebral infarcts (<20 mm). None of the patients had history of congestive heart failure or presented an ejection fraction <40% of the echocardiogram. Accordingly, Cheyne-Stokes respiration in patients with lacunar stroke can be reasonably considered secondary to the acute lacunar infarction.

 Sleep-related breathing disorders have been increasingly recognized as a risk factor for stroke and, on the other hand, acute stroke may be the cause of either central apneas or Cheyne-Stokes respiration. In the study of Parra et al. [11] of sleep-related breathing disorders in stroke patients, it was shown that during the stable phase of stroke, there was a decrease of respiratory events mostly of central apneas, whereas obstructive events remain unaltered. Also, acute stroke may cause destabilization of the upper airways or according to the cerebral area affected, motor weakness of the muscles of the upper respiratory tract favouring obstructive apneas or hypopneas.

 One question to be solved in these types of studies is the cutoff value for central apnea and Cheyne-Stokes respiration that may have clinical, functional, or prognostic impact. Most authors have considered a central apnea index >5 as the minimum number of central apneas per hour that might have some clinical repercussion, although this cutoff value is still undefined [8]. Moreover, central hypopneas are not included in this index given the difficulty in distinguishing obstructive hypopneas from central hypopneas in polysomnographic studies [21]. In relation to Cheyne-Stokes respiration, 10% was the minimum time that may have some clinical relevance rather than whether or not Cheyne-Stokes respiration was observed. In the present study, lacunar stroke patients with Cheyne-Stokes respiration showed a significantly higher mean AHI value than patients with a normal breathing pattern despite the fact that differences in BMI between both groups were not found. The central apnea index was also significantly higher among patients with Cheyne-Stokes respiration but without affecting daytime sleepiness, although with a lower nocturnal SaO_2_ probably at the expense of obstructive phenomena.

 It should be noted that significant differences in demographic data, cardiovascular risk factors, and clinical features between patients with and without Cheyne-Stokes respiration were not documented. The pons and the internal capsule were the most common topographies in both study groups. Up to the present time, a relationship between Cheyne-Stokes respiration and a particular cerebral topography could not have been established [13]. Although pure motor hemiparesis was the most frequent lacunar syndrome, a relationship between type of lacunar syndrome and presence of Cheyne-Stokes respiration was not found. However, none of the patients with pure sensory stroke had Cheyne-Stokes respiration as compared with 12.2% among patients without Cheyne-Stokes respiration, a fact that may suggest a higher probability of Cheyne-Stokes respiration in lacunar syndromes with motor dysfunction due to a lesion of the pyramidal tract. This observation can also be related to a higher mean AHI in the group of patients with Cheyne-Stokes respiration mainly related to obstructive sleep apneas prior to the stroke, but also to obstructive phenomena caused by destabilization of the upper airways or to involvement of pyramidal-related musculature that without affecting swallowing (none of the patients required placement of a nasogastric tube) may cause a higher instability of the upper respiratory tract during the night.

 The small sample size is the main limitation of the study. Although the number of patients was insufficient to assess the impact of Cheyne-Stokes respiration on the early outcome of lacunar stroke (a stroke subtype with mild neurological impairment [22]), a trend towards a more severe stroke and worse prognosis in patients with Cheyne-Stokes respiration was found. Different studies have shown a less favourable immediate and long-term clinical course and worse quality of life in stroke patients with sleep-related breathing disorders [20–26], although some authors have suggested that sleep-related breathing disorders would most affect the time of stroke recovery [27]. In our study, the mean scores of the Barthel index, the Canadian Neurological scale, and the modified Rankin scale between patients with and without Cheyne-Stokes respiration were similar, but further studies with a larger sample size and sufficient statistical power are needed to assess the impact of Cheyne-Stokes respiration in lacunar infarction. The use of a portable respiratory recording device instead of a full-night polysomnography may be considered a limitation of the study. However, this procedure has been previously validated using full polysomnography and has been used in stroke patients [11].

 In summary, Cheyne-Stokes respiration was documented in 20.6% of patients with lacunar stroke. The presence of Cheyne-Stokes respiration appeared to be related to a trend towards a higher functional stroke severity and worse prognosis. Our findings warrant further investigation in a larger study population.

## Figures and Tables

**Table 1:** Clinical characteristics of 68 patients with lacunar stroke according to the presence of Cheyne-Stokes respiration.

Variables	Cheyne-Stokes respiration		*P* value
	Present, *n* = 14	Absent, *n* = 54	
Sex, men/women	9/5	28/26	0.405
Age, years, mean (SD)	76.7 (6.8)	72.3 (10.0)	0.101
Body mass index, BMI, kg/m^2^, mean (SD)	26.3 (4.6)	26.2 (3.2)	0.788
Respiratory data, mean (SD)			
AHI	34.9 (21.7)	18.5 (14.4)	0.001
Central apnea index	13.1 (13.8)	1.8 (3.4)	0.000
Obstructive events	21.8 (12.7)	16.7 (13.9)	0.390
Epworth sleepiness scale	3.3 (2.6)	5.4 (3.4)	0.064
CT90(%)	8.4 (11.3)	2.5 (5.2)	0.110
Vascular risk factors			
Hypertension	11 (78.6)	34 (63)	0.271
Diabetes mellitus	4 (28.6)	22 (40.7)	0.404
Previous TIA	3 (21.4)	4 (7.4)	0.124
Atrial fibrillation	1 (7.1)	6 (11.1)	0.633
Ischemic heart disease	0	2 (3.7)	0.465
Peripheral artery disease	1 (7.1)	4 (7.4)	0.973
Chronic obstructive pulmonary disease	1 (7.1)	2 (3.7)	0.577
Dyslipidemia	4 (28.6)	8 (14.8)	0.618
Smoking	4 (28.6)	8 (14.8)	0.229
Alcohol abuse	0	3 (5.6)	0.367
Salient clinical features			
Sudden onset	6 (42.9)	31 (57.4)	0.330
Headache	2 (14.3)	7 (13)	0.896
Motor deficit	11 (78.6)	37 (68.5)	0.462
Sensory deficit	4 (28.6)	20 (37)	0.555
Speech disturbances	7 (50)	35 (64.8)	0.309
Lacunar syndrome			0.163
Pure motor hemiparesis	6 (42.9)	24 (44.4)	0.915
Pure sensory stroke	0	12 (12.2)	0.121
Sensorimotor stroke	3 (21.4)	3 (5.6)	0.181
Ataxic hemiparesis + dysarthria clumsy hand	3 (21.4)	9 (16.7)	0.982
Atypical syndrome	2 (14.3)	6 (11.1)	1.0
Topography of lacunes			
Internal capsule	5 (37.5)	21 (38.9)	0.828
Centrum semiovale	3 (21.4)	4 (7.4)	0.124
Basal ganglia	0	6 (11.1)	0.191
Thalamus	1 (7.1)	11 (20.4)	0.247
Pons	5 (35.7)	14 (25.9)	0.467

Data as numbers and percentages in parenthesis unless otherwise stated.

**Table 2:** Outcome results in 68 patients with lacunar stroke according to the presence of Cheyne-Stokes respiration.

Variables	Cheyne-Stokes respiration		*P* value
	Present, *n* = 14	Absent, *n* = 54	
Barthel index	58.3 (32.5)	75.8 (27.9)	0.130
Canadian Neurological scale	6.9 (1.9)	7.9 (2.1)	0.131
Modified Rankin scale	2.4 (1.3)	2.2 (1.1)	0.131
In-hospital mortality	0	0	
Length of hospital stay	14.9 (11.1)	11.5 (5.6)	0.184

Data as mean (standard deviation, SD).
